# 4,4′-Diiodo-2,2′-[(3a*R*,7a*R*)-2,3,3a,4,5,6,7,7a-octa­hydro-1*H*-1,3-benzimidazole-1,3-di­yl)bis­(methyl­ene)]diphenol

**DOI:** 10.1107/S1600536811030054

**Published:** 2011-08-06

**Authors:** Augusto Rivera, Diego Quiroga, Jaime Ríos-Motta, Karla Fejfarová, Michal Dušek

**Affiliations:** a Departamento de Química, Universidad Nacional de Colombia, Ciudad Universitaria, Bogotá, Colombia; b Institute of Physics ASCR, v.v.i., Na Slovance 2, 182 21 Praha 8, Czech Republic

## Abstract

In the crystal structure of the title compound, C_21_H_24_I_2_N_2_O_2_, the two N atoms of the imidazolidine moiety are linked to the hy­droxy groups by intra­molecular O—H⋯N hydrogen-bonding inter­actions. The cyclo­hexane ring adopts a chair conformation and the heterocyclic ring to which it is fused has a twisted envelope conformation.

## Related literature

For related structures, see: Rivera *et al.* (2010 ▶, 2011*a* ▶,*b* ▶); Merz (2006 ▶).
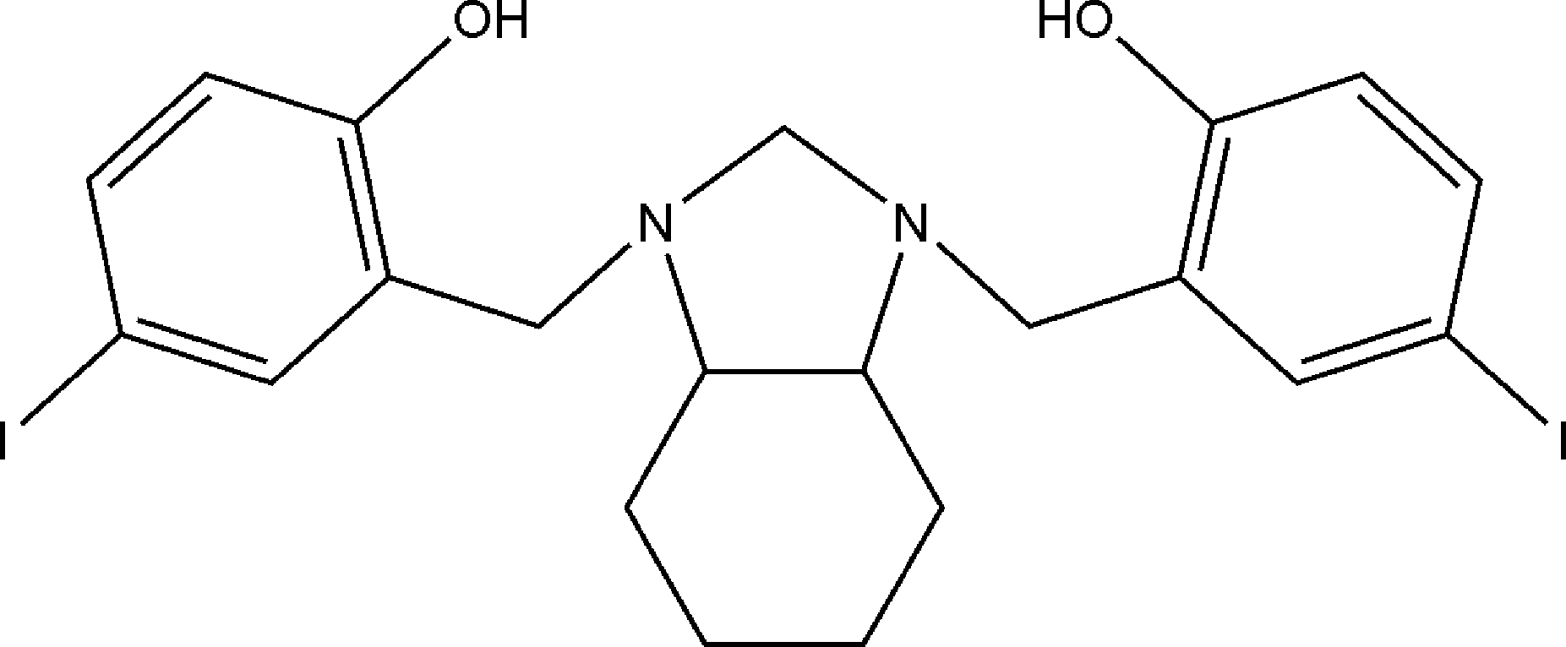


## Experimental

### 

#### Crystal data

C_21_H_24_I_2_N_2_O_2_
*M* *_r_* = 590.2
Monoclinic, 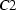

*a* = 24.5822 (12) Å
*b* = 6.1121 (3) Å
*c* = 16.5557 (10) Å
β = 121.119 (6)°
*V* = 2129.5 (2) Å^3^
*Z* = 4
Cu *K*α radiation
μ = 23.34 mm^−1^
*T* = 120 K
0.26 × 0.12 × 0.05 mm

#### Data collection

Agilent Xcalibur diffractometer with an Atlas (Gemini Ultra Cu) detector
Absorption correction: analytical (*CrysAlis PRO*; Agilent, 2010 ▶) *T*_min_ = 0.074, *T*_max_ = 0.424
11449 measured reflections
3650 independent reflections
3397 reflections with *I* > 3σ(*I*)
*R*_int_ = 0.062

#### Refinement

*R*[*F* ^2^ > 2σ(*F* ^2^)] = 0.040
*wR*(*F* ^2^) = 0.106
*S* = 1.30
3650 reflections
250 parameters
2 restraints
H atoms treated by a mixture of independent and constrained refinement
Δρ_max_ = 0.85 e Å^−3^
Δρ_min_ = −0.87 e Å^−3^
Absolute structure: Flack (1983 ▶), 1566 Friedel pairs
Flack parameter: 0.079 (13)

### 

Data collection: *CrysAlis PRO* (Agilent, 2010 ▶); cell refinement: *CrysAlis PRO*; data reduction: *CrysAlis PRO*; program(s) used to solve structure: *SIR2002* (Burla *et al.*, 2003 ▶); program(s) used to refine structure: *JANA2006* (Petříček *et al.*, 2006 ▶); molecular graphics: *DIAMOND* (Brandenburg & Putz, 2005 ▶); software used to prepare material for publication: *JANA2006*.

## Supplementary Material

Crystal structure: contains datablock(s) global, I. DOI: 10.1107/S1600536811030054/nc2238sup1.cif

Structure factors: contains datablock(s) I. DOI: 10.1107/S1600536811030054/nc2238Isup2.hkl

Supplementary material file. DOI: 10.1107/S1600536811030054/nc2238Isup3.cml

Additional supplementary materials:  crystallographic information; 3D view; checkCIF report

## Figures and Tables

**Table 1:** Hydrogen-bond geometry (Å, °)

*D*—H⋯*A*	*D*—H	H⋯*A*	*D*⋯*A*	*D*—H⋯*A*
O1—H1O⋯N1	0.84 (10)	1.90 (7)	2.672 (9)	152 (11)
O2—H2O⋯N2	0.84 (8)	1.91 (8)	2.686 (9)	154 (8)

**Fig. 1.**
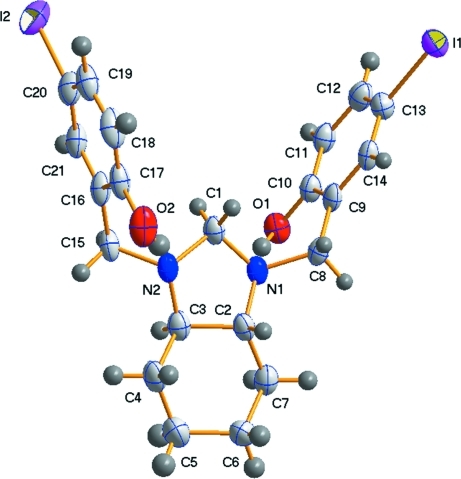
A view of (I) with the numbering scheme. Displacement ellipsoids are drawn at the 50% probability level.

## supplementary crystallographic information

### Comment

In our investigations we have obtained a new family of Mannich bases from the aminal cage (2*R*,7*R*,11*S*,16S)-1,8,10,17-tetraazapentacyclo- [8.8.1.1^8,17^0.^2,7^0.^11,16^]icosane and *p*-halophenols (*p*-XPhOH) where the *p*-substituent in the aromatic ring was Cl, Br or F (Rivera, *et al.* 2010, 2011*a*, 2011*b*). The X-ray diffraction analyses suggested an influence of resonance and inductive effects in the strength of hydrogen bonding interaction. To complete the halogen series, we report here the synthesis and crystal structure of the title compound (I). The molecular structure and atom-numbering scheme for (I) are shown in Fig. 1. Its X-ray structure confirms the presence of intramolecular hydrogen bonds between the phenolic hydroxyl groups and nitrogen atoms (Table 1) which are longer in comparison with related structures (Rivera, *et al.* 2010, 2011*a*). The observed N···O distances and the observed C–O bond lengths [1.363 (12) Å and 1.372 (10) Å] are longer in relation to the *p*-chloro and *p*-bromo related structures, but these values are in a good agreement with the *p*-fluoro derivative (Rivera *et al.* 2011*b*). These results indicate a decrease in hydrogen-bonding strength due to the presence of iodine atom, which is the less electronegative atom. The C—I bond lengths (I1—C13, 2.105 (10) Å; I2—C20, 2.108 (8) Å) are in good agreement with the value reported for iodophenols (2-iodophenol, 2.078 (9) Å; 3-iodophenol, 2.109 (5) Å; 4-iodophenol, 2.104 (5) Å; Merz, 2006). There are endocyclic angle distortions on the aromatic ring, which are associated with electron-withdrawing substituent and electron releasing substituent effect. The slightly enlarged C12—C13—C14 and C19—C20—C21 angles are a few degrees larger than 120°, showing an effect of the iodine group on benzene ring geometries. The endocyclic C10—C9—C14 and C17—C16—C21 angle values are few degrees less than 120° (where there are *o*-aminomethylene groups on C9 and C16). The cyclohexanediamine fragment adopts a chair conformation showing intraanular C—C—C bond angle values in the range of 107.9 (8) ° to 112.5 (6) ° which are close to normal tetrahedral bond angles. The nitrogen lone pairs are oriented in an anti-axial conformation; therefore the heterocyclic ring adopts a twisted envelope conformation.

### Experimental

*Physical Measurements*

The melting point was determined with an Electrothermal apparatus. NMR spectra were performed in CDCl_3_ at room temperature on a Bruker AMX 400 Advance spectrometer.

*Preparation of 4,4'-Diiodo-2,2'-{[(3aR,7aR)-2,3,3a,4,5,6,7,7a-octahydro-1H-1,3- benzimidazole-1,3-diyl)] bis(methylene)]}diphenol) (I)*

A solution of *p*-iodophenol (440 mg, 2.00 mmol) in dioxane (3 ml) was added dropwise to (2*R*,7*R*,11*S*,16S)-1,8,10,17-tetraazapenta- cyclo[8.8.1.1^8,17^.0^2,7^.0^11,16^]icosane (276 mg, 1.00 mmol) in dioxane (3 ml) and water (4 ml). The mixture was refluxed for about 6 h. The solvent was evaporated under reduced pressure until a sticky residue appeared. The product was purified by chromatography on a silica column, and subjected to gradient elution with benzene:ethyl acetate (yield 25%, m.p. = 477–479 K). The crude product (100 mg, 0.169 mmol) was dissolved in 5 ml of a 4:1 mixture of chloroform: methanol. Single crystals of the title compound (I) suitable for X-ray analysis were grown by slow evaporation of the solvent from a chloroform:methanol mixture at room temperature over a period of about 2 weeks. (yield 35%). ^1^H NMR (CDCl_3_, 400 MHz): δ 1.29 (4*H*, m), 1.85 (2*H*, m), 2.05 (2*H*, m), 2.32 (2*H*, m), 3.39 (2*H*, d, *^2^J* = 14.0 Hz, ArCH_2_N), 3.50 (2*H*, s, NCH_2_N), 4.13 (2*H*, d, *^2^J* = 14.0 Hz, ArCH_2_N), 6.59 (2*H*, d, *^3^J* = 8.4 Hz), 7.23 (2*H*, s), 7.43 (2*H*, d, *^3^J* = 8.4 Hz), 10.57 (2*H*, bs, ArOH). ^13^C NMR (CDCl_3_, 100 MHz): δ 23.9, 28.8, 55.6, 69.0, 75.7, 80.8, 118.6, 124.0, 136.5, 137.8, 157.3.

### Refinement

The hydrogen attached to C atoms were positioned geometrically and kept in ideal positions with C–H distance 0.96 Å during the refinement. The hydroxyl hydrogen atoms were found in difference Fourier maps and refined with a distance restraint d(O—H) = 0.84 (2) Å. The isotropic atomic displacement parameters of hydrogen atoms were evaluated as 1.2×*U*_eq_ of the parent atom. The absolute structure was determined on the basis of 1566 Friedel pairs.

### Crystal data

**Table:**

C_21_H_24_I_2_N_2_O_2_	*F*(000) = 1144
*M**_r_* = 590.2	*D*_x_ = 1.840 Mg m^−^^3^
Monoclinic, *C*2	Cu *K*α radiation, λ = 1.5418 Å
Hall symbol: C 2y	Cell parameters from 5885 reflections
*a* = 24.5822 (12) Å	θ = 3.1–67.1°
*b* = 6.1121 (3) Å	µ = 23.34 mm^−^^1^
*c* = 16.5557 (10) Å	*T* = 120 K
β = 121.119 (6)°	Prism, colourless
*V* = 2129.5 (2) Å^3^	0.26 × 0.12 × 0.05 mm
*Z* = 4	

### Data collection

**Table:**

Agilent Xcalibur diffractometer with an Atlas (Gemini ultra Cu) detector	3650 independent reflections
Radiation source: Enhance Ultra (Cu) X-ray Source	3397 reflections with *I* > 3σ(*I*)
mirror	*R*_int_ = 0.062
Detector resolution: 10.3784 pixels mm^-1^	θ_max_ = 67.2°, θ_min_ = 3.1°
Rotation method data acquisition using ω scans	*h* = −29→29
Absorption correction: analytical (*CrysAlis PRO*; Agilent, 2010)	*k* = −7→7
*T*_min_ = 0.074, *T*_max_ = 0.424	*l* = −19→19
11449 measured reflections	

### Refinement

**Table:**

Refinement on *F*^2^	H atoms treated by a mixture of independent and constrained refinement
*R*[*F*^2^ > 2σ(*F*^2^)] = 0.040	Weighting scheme based on measured s.u.'s *w* = 1/[σ^2^(*I*) + 0.0016*I*^2^]
*wR*(*F*^2^) = 0.106	(Δ/σ)_max_ = 0.038
*S* = 1.30	Δρ_max_ = 0.85 e Å^−^^3^
3650 reflections	Δρ_min_ = −0.87 e Å^−^^3^
250 parameters	Absolute structure: Flack (1983), 1566 Friedel pairs
2 restraints	Flack parameter: 0.079 (13)
91 constraints	

### Special details

**Table:**

Experimental. CrysAlis Pro (Agilent, 2010), Analytical numeric absorption correction using a multifaceted crystal model.
Refinement. The refinement was carried out against all reflections. The conventional *R*-factor is always based on *F*. The goodness of fit as well as the weighted *R*-factor are based on *F* and *F*^2^ for refinement carried out on *F* and *F*^2^, respectively. The threshold expression is used only for calculating *R*-factors *etc*. and it is not relevant to the choice of reflections for refinement.The program used for refinement, Jana2006, uses the weighting scheme based on the experimental expectations, see _refine_ls_weighting_details, that does not force *S* to be one. Therefore the values of *S* are usually larger than the ones from the *SHELX* program.The absolute structure was determined on the basis of 1566 Friedel pairs (Flack, 1983),

### Fractional atomic coordinates and isotropic or equivalent isotropic displacement parameters (Å^2^)

**Table:**

	*x*	*y*	*z*	*U*_iso_*/*U*_eq_	
I1	0.40926 (2)	0.38885	0.03435 (3)	0.0457 (2)	
I2	0.54564 (3)	0.34180 (15)	0.67075 (4)	0.0621 (3)	
O1	0.2558 (2)	0.8312 (10)	0.1940 (3)	0.040 (2)	
O2	0.2940 (3)	−0.1604 (11)	0.4035 (4)	0.054 (3)	
N1	0.2165 (3)	0.4306 (11)	0.2051 (4)	0.034 (2)	
N2	0.2436 (3)	0.2401 (11)	0.3459 (4)	0.035 (3)	
C1	0.2707 (3)	0.3359 (13)	0.2912 (4)	0.034 (3)	
C2	0.1598 (3)	0.3332 (14)	0.1992 (4)	0.036 (3)	
C3	0.1790 (3)	0.3314 (15)	0.3014 (5)	0.038 (3)	
C4	0.1317 (4)	0.2045 (16)	0.3154 (6)	0.045 (4)	
C5	0.0666 (4)	0.314 (2)	0.2559 (6)	0.061 (5)	
C6	0.0474 (3)	0.335 (2)	0.1518 (5)	0.051 (4)	
C7	0.0975 (4)	0.4517 (15)	0.1400 (5)	0.042 (3)	
C8	0.2198 (3)	0.3905 (16)	0.1187 (4)	0.031 (3)	
C9	0.2733 (3)	0.5131 (13)	0.1224 (5)	0.034 (3)	
C10	0.2890 (4)	0.7266 (14)	0.1603 (5)	0.035 (3)	
C11	0.3386 (3)	0.8414 (15)	0.1623 (4)	0.037 (3)	
C12	0.3732 (4)	0.7461 (14)	0.1254 (5)	0.042 (3)	
C13	0.3568 (4)	0.5396 (14)	0.0869 (5)	0.037 (3)	
C14	0.3084 (3)	0.4225 (12)	0.0860 (4)	0.031 (3)	
C15	0.2835 (4)	0.2855 (14)	0.4477 (5)	0.038 (3)	
C16	0.3459 (4)	0.1688 (14)	0.4877 (5)	0.038 (3)	
C17	0.3489 (4)	−0.0472 (14)	0.4615 (5)	0.042 (4)	
C18	0.4071 (4)	−0.1506 (18)	0.4971 (6)	0.052 (4)	
C19	0.4628 (5)	−0.0464 (14)	0.5584 (6)	0.046 (4)	
C20	0.4608 (4)	0.1693 (16)	0.5825 (6)	0.047 (4)	
C21	0.4035 (4)	0.2758 (15)	0.5479 (5)	0.041 (3)	
H1a	0.30018	0.449807	0.327486	0.041*	
H1b	0.289698	0.222021	0.273989	0.041*	
H2	0.150156	0.194368	0.167635	0.0427*	
H3	0.179095	0.470059	0.328724	0.046*	
H4a	0.144224	0.208887	0.380735	0.0534*	
H4b	0.129369	0.056584	0.294365	0.0534*	
H5a	0.067702	0.456816	0.281037	0.0736*	
H5b	0.035132	0.23068	0.260104	0.0736*	
H6a	0.039828	0.192423	0.123906	0.0611*	
H6b	0.007888	0.412918	0.117567	0.0611*	
H7a	0.085623	0.447024	0.074901	0.0506*	
H7b	0.101731	0.600035	0.161522	0.0506*	
H8a	0.22518	0.236821	0.113022	0.0373*	
H8b	0.180562	0.434385	0.06391	0.0373*	
H11	0.349133	0.985749	0.188885	0.0443*	
H12	0.40771	0.823632	0.127037	0.05*	
H14	0.298725	0.277391	0.060112	0.0371*	
H15a	0.290776	0.440117	0.457354	0.0451*	
H15b	0.26204	0.236075	0.479059	0.0451*	
H18	0.408329	−0.298327	0.478383	0.0625*	
H19	0.502706	−0.121714	0.584329	0.0552*	
H21	0.402993	0.425237	0.565416	0.0489*	
H1O	0.240 (4)	0.728 (11)	0.209 (7)	0.0482*	
H2O	0.270 (4)	−0.059 (13)	0.370 (7)	0.0643*	

### Atomic displacement parameters (Å^2^)

**Table:**

	*U*^11^	*U*^22^	*U*^33^	*U*^12^	*U*^13^	*U*^23^
I1	0.0504 (2)	0.0491 (3)	0.0454 (2)	−0.0022 (2)	0.0304 (2)	−0.0027 (2)
I2	0.0572 (3)	0.0623 (5)	0.0429 (3)	0.0124 (3)	0.0089 (2)	−0.0038 (3)
O1	0.054 (3)	0.030 (3)	0.042 (2)	−0.003 (2)	0.028 (2)	−0.001 (2)
O2	0.090 (4)	0.025 (4)	0.047 (3)	−0.007 (3)	0.036 (3)	−0.004 (3)
N1	0.045 (3)	0.031 (4)	0.029 (3)	−0.005 (2)	0.021 (2)	−0.004 (2)
N2	0.048 (3)	0.032 (4)	0.026 (3)	−0.007 (3)	0.020 (3)	−0.003 (2)
C1	0.045 (3)	0.030 (4)	0.031 (3)	−0.005 (3)	0.022 (3)	0.000 (3)
C2	0.045 (3)	0.035 (5)	0.033 (3)	−0.008 (3)	0.025 (3)	−0.003 (3)
C3	0.050 (4)	0.033 (5)	0.034 (3)	−0.005 (3)	0.024 (3)	−0.001 (3)
C4	0.058 (4)	0.046 (5)	0.039 (4)	−0.011 (4)	0.031 (4)	0.000 (4)
C5	0.057 (5)	0.088 (9)	0.047 (4)	−0.008 (5)	0.033 (4)	−0.001 (5)
C6	0.042 (4)	0.070 (7)	0.044 (4)	−0.009 (4)	0.025 (3)	−0.006 (5)
C7	0.051 (4)	0.043 (5)	0.034 (4)	−0.004 (3)	0.022 (3)	0.000 (3)
C8	0.039 (3)	0.023 (4)	0.026 (3)	−0.001 (3)	0.013 (2)	0.006 (3)
C9	0.043 (4)	0.034 (5)	0.022 (3)	0.000 (3)	0.015 (3)	0.007 (3)
C10	0.041 (4)	0.034 (5)	0.028 (3)	−0.004 (3)	0.016 (3)	−0.003 (3)
C11	0.047 (3)	0.029 (5)	0.029 (3)	−0.005 (3)	0.016 (3)	−0.003 (3)
C12	0.046 (4)	0.040 (5)	0.034 (4)	−0.006 (3)	0.017 (3)	0.004 (3)
C13	0.043 (4)	0.040 (5)	0.033 (4)	−0.001 (3)	0.022 (3)	−0.001 (3)
C14	0.046 (3)	0.020 (4)	0.023 (3)	−0.004 (3)	0.015 (3)	0.000 (3)
C15	0.059 (4)	0.031 (5)	0.029 (3)	0.005 (3)	0.028 (3)	0.001 (3)
C16	0.062 (5)	0.025 (4)	0.028 (4)	0.003 (3)	0.024 (4)	0.002 (3)
C17	0.075 (5)	0.025 (5)	0.033 (4)	−0.004 (4)	0.034 (4)	−0.003 (3)
C18	0.092 (6)	0.028 (6)	0.048 (4)	0.008 (5)	0.044 (4)	−0.003 (4)
C19	0.078 (6)	0.031 (5)	0.041 (4)	0.014 (4)	0.039 (4)	0.009 (3)
C20	0.060 (5)	0.043 (5)	0.034 (4)	0.013 (4)	0.023 (4)	0.009 (4)
C21	0.063 (5)	0.032 (5)	0.027 (3)	0.006 (3)	0.023 (3)	0.003 (3)

### Geometric parameters (Å, °)

**Table:**

I1—C13	2.105 (10)	C6—H6b	0.96
I2—C20	2.108 (8)	C7—H7a	0.96
O1—C10	1.363 (12)	C7—H7b	0.96
O1—H1O	0.84 (10)	C8—C9	1.488 (12)
O2—C17	1.372 (10)	C8—H8a	0.96
O2—H2O	0.84 (8)	C8—H8b	0.96
N1—C1	1.476 (7)	C9—C10	1.412 (11)
N1—C2	1.471 (11)	C9—C14	1.396 (13)
N1—C8	1.494 (11)	C10—C11	1.392 (13)
N2—C1	1.494 (12)	C11—C12	1.405 (14)
N2—C3	1.473 (10)	C11—H11	0.96
N2—C15	1.472 (9)	C12—C13	1.377 (12)
C1—H1a	0.96	C12—H12	0.96
C1—H1b	0.96	C13—C14	1.383 (12)
C2—C3	1.503 (11)	C14—H14	0.96
C2—C7	1.510 (10)	C15—C16	1.501 (12)
C2—H2	0.96	C15—H15a	0.96
C3—C4	1.511 (14)	C15—H15b	0.96
C3—H3	0.96	C16—C17	1.404 (12)
C4—C5	1.532 (12)	C16—C21	1.402 (11)
C4—H4a	0.96	C17—C18	1.385 (14)
C4—H4b	0.96	C18—C19	1.370 (12)
C5—C6	1.539 (13)	C18—H18	0.96
C5—H5a	0.96	C19—C20	1.385 (13)
C5—H5b	0.96	C19—H19	0.96
C6—C7	1.520 (15)	C20—C21	1.380 (13)
C6—H6a	0.96	C21—H21	0.96
			
C10—O1—H1O	104 (7)	H7a—C7—H7b	110.9091
C17—O2—H2O	101 (6)	N1—C8—C9	111.3 (6)
C1—N1—C2	104.8 (6)	N1—C8—H8a	109.4702
C1—N1—C8	113.0 (6)	N1—C8—H8b	109.4715
C2—N1—C8	112.9 (5)	C9—C8—H8a	109.4718
C1—N2—C3	104.6 (6)	C9—C8—H8b	109.4709
C1—N2—C15	112.3 (6)	H8a—C8—H8b	107.5975
C3—N2—C15	114.1 (7)	C8—C9—C10	121.1 (9)
N1—C1—N2	106.1 (6)	C8—C9—C14	120.8 (7)
N1—C1—H1a	109.4714	C10—C9—C14	118.1 (8)
N1—C1—H1b	109.4713	O1—C10—C9	122.1 (8)
N2—C1—H1a	109.472	O1—C10—C11	117.2 (7)
N2—C1—H1b	109.471	C9—C10—C11	120.7 (9)
H1a—C1—H1b	112.6582	C10—C11—C12	120.0 (8)
N1—C2—C3	101.2 (5)	C10—C11—H11	119.9796
N1—C2—C7	117.1 (7)	C12—C11—H11	119.9777
N1—C2—H2	110.6437	C11—C12—C13	118.9 (9)
C3—C2—C7	110.9 (8)	C11—C12—H12	120.5572
C3—C2—H2	116.8819	C13—C12—H12	120.5582
C7—C2—H2	100.8307	I1—C13—C12	119.8 (7)
N2—C3—C2	101.3 (7)	I1—C13—C14	118.5 (6)
N2—C3—C4	116.5 (7)	C12—C13—C14	121.6 (9)
N2—C3—H3	111.0115	C9—C14—C13	120.7 (7)
C2—C3—C4	111.0 (6)	C9—C14—H14	119.6736
C2—C3—H3	116.5093	C13—C14—H14	119.6733
C4—C3—H3	101.3338	N2—C15—C16	109.9 (8)
C3—C4—C5	107.9 (8)	N2—C15—H15a	109.4717
C3—C4—H4a	109.4721	N2—C15—H15b	109.4717
C3—C4—H4b	109.4725	C16—C15—H15a	109.4703
C5—C4—H4a	109.4701	C16—C15—H15b	109.471
C5—C4—H4b	109.47	H15a—C15—H15b	109.0826
H4a—C4—H4b	111.0366	C15—C16—C17	121.1 (7)
C4—C5—C6	111.8 (9)	C15—C16—C21	121.2 (8)
C4—C5—H5a	109.4716	C17—C16—C21	117.6 (8)
C4—C5—H5b	109.4713	O2—C17—C16	120.0 (8)
C6—C5—H5a	109.4709	O2—C17—C18	119.6 (8)
C6—C5—H5b	109.4715	C16—C17—C18	120.4 (8)
H5a—C5—H5b	107.0854	C17—C18—C19	121.3 (10)
C5—C6—C7	112.5 (6)	C17—C18—H18	119.3262
C5—C6—H6a	109.4704	C19—C18—H18	119.324
C5—C6—H6b	109.4702	C18—C19—C20	119.0 (9)
C7—C6—H6a	109.4723	C18—C19—H19	120.5016
C7—C6—H6b	109.4718	C20—C19—H19	120.5014
H6a—C6—H6b	106.2371	I2—C20—C19	120.4 (7)
C2—C7—C6	108.0 (7)	I2—C20—C21	118.9 (7)
C2—C7—H7a	109.4703	C19—C20—C21	120.7 (8)
C2—C7—H7b	109.4705	C16—C21—C20	120.9 (8)
C6—C7—H7a	109.4719	C16—C21—H21	119.5344
C6—C7—H7b	109.4721	C20—C21—H21	119.5335
			
?—?—?—?	?		

### Hydrogen-bond geometry (Å, °)

**Table:**

*D*—H···*A*	*D*—H	H···*A*	*D*···*A*	*D*—H···*A*
O1—H1O···N1	0.84 (10)	1.90 (7)	2.672 (9)	152 (11)
O2—H2O···N2	0.84 (8)	1.91 (8)	2.686 (9)	154 (8)
